# Genomic Relatedness, Antibiotic Resistance and Virulence Traits of *Campylobacter jejuni* HS19 Isolates From Cattle in China Indicate Pathogenic Potential

**DOI:** 10.3389/fmicb.2021.783750

**Published:** 2021-11-30

**Authors:** Xiaoqi Zang, Pingyu Huang, Jie Li, Xinan Jiao, Jinlin Huang

**Affiliations:** ^1^Jiangsu Key Laboratory of Zoonosis, Jiangsu Co-Innovation Center for Prevention and Control of Important Animal Infectious Diseases and Zoonoses, Yangzhou University, Yangzhou, China; ^2^Key Laboratory of Prevention and Control of Biological Hazard Factors (Animal Origin) for Agrifood Safety and Quality, Ministry of Agriculture of China, Yangzhou, China; ^3^Joint International Research Laboratory of Agriculture and Agri-Product Safety, Ministry of Education of China, Yangzhou, China

**Keywords:** *Campylobacter jejuni* isolates from cattle, serotype HS19, zoonotic hazard, whole-genome sequencing, phylogenetic relatedness, IL-10^–/–^ C57BL/6 mice

## Abstract

Although campylobacteriosis is a zoonotic foodborne illness, high-risk isolates from animal sources are rarely characterized, and the pathogenic potential of zoonotic strains remains an obstacle to effective intervention against human infection. HS19 has been acknowledged as a maker serotype represented by *Campylobacter jejuni* (*C. jejuni*) isolates from patients with post-infection Guillain-Barré syndrome (GBS), which is circulation in developed countries. However, a previous serotype epidemiological study of *C. jejuni* isolates in an animal population revealed that HS19 was also prevalent in isolates from cattle in China. In this study, to investigate the hazardous potential of zoonotic strains, 14 HS19 isolates from cattle were systematically characterized both by genotype and phenotype. The results showed that all of these cattle isolates belonged to the ST-22 complex, a high-risk lineage represented by 77.2% HS19 clinical isolates from patients worldwide in the PubMLST database, indicating that the ST-22 complex is the prominent clonal complex of HS19 isolates, as well as the possibility of clonal spread of HS19 isolates across different regions and hosts. Nevertheless, these cattle strains clustered closely with the HS19 isolates from patients, suggesting a remarkable phylogenetic relatedness and genomic similarity. Importantly, both tetracycline genes *tet(O)* and *gyrA* (T86I) reached a higher proportional representation among the cattle isolates than among the human clinical isolates. A worrying level of multidrug resistance (MDR) was observed in all the cattle isolates, and two MDR profiles of the cattle isolates also existed in human clinical isolates. Notably, although shared with the same serotype HS19 and sequence type ST-22, 35.7% of cattle isolates induced severe gastrointestinal pathology in the IL-10^–/–^ C57BL/6 mice model, indicating that some bacteria could change due to host adaptation to induce a disease epidemic, thus the associated genetic elements deserve further investigation. In this study, HS19 isolates from cattle were first characterized by a systematic evaluation of bacterial genomics and *in vitro* virulence, which improved our understanding of the potential zoonotic hazard from food animal isolates with high-risk serotypes, and provided critical information for the development of targeted *C. jejuni* mitigation strategies.

## Introduction

*Campylobacter jejuni* is the leading cause of bacterial foodborne gastroenteritis in humans, both in developed and developing countries (Ruiz-Palacios, 2007; Kaakoush et al., 2015), making it a great threat to the public’s health. While the majority of cases are self-limiting, they can spread into the bloodstream in immunocompromised individuals and become potentially lethal (Whitehouse et al., 2018). In some instances, affected patients are at risk of Guillain-Barré syndrome (GBS), a severe post-infectious autoimmune disease that occurs weeks or months after the initial infectious gastrointestinal manifestation (Pithadia and Kakadia, 2010), which can also sometimes be life-threatening (Nachamkin, 2002). The high incidence of *C. jejuni-*associated disease in humans is largely due to its prevalence as a zoonotic agent in animals (Sheppard et al., 2011; Burnham and Hendrixson, 2018). As a part of the commensal microbiota of numerous host species, fecal contamination from carrier animals is considered to be the primary source of *C. jejuni* (Koenraad et al., 1997; Guirado et al., 2020). Chicken is a common source of *C. jejuni* in sporadic infection, and the role of cattle is also notable (Hakkinen et al., 2009). Significant associations emerged between certain clonal complexes from human infection and the contact with cattle, the consumption of unpasteurized milk and raw minced meat, raising the question about the pathogenic potential of cattle isolates (Kärenlampi et al., 2007; Wilson et al., 2008; Costard et al., 2017; Hsu et al., 2020). In fact, not all strains or genetic lineages pose equal risks to human health. Although campylobacteriosis is a zoonotic foodborne disease, the majority of the reported high-risk strains were from clinical patients, and isolates from animal sources have rarely been characterized.

The identification and profiling of *C. jejuni* virulence determinants are crucial for the risk assessment of campylobacteriosis infection (Fiedoruk et al., 2019), whereas gaining insight into the distribution of virulence-associated genes among strains might shed some light on the mechanisms exploited by *Campylobacter* to trigger infection (Iglesias-Torrens et al., 2018). Notably, *C. jejuni* does not possess numerous classical virulence factors. Cytolethal distending enterotoxin (Cdt) is the only virulence determinant located on the *C. jejuni* chromosome, however, its role in pathogenesis is still unclear (Burnham and Hendrixson, 2018). Moreover, the self-limiting feature of most campylobacteriosis cases, as well as the lack of a traceable animal model, hinder the hazard evaluation of *Campylobacter* species (Crofts et al., 2018). Multiple bacterial factors have been implicated in the pathogenesis of campylobacteriosis supporting *Campylobacter* to invade the host and evade the host’s defenses (de Oliveira et al., 2019). Capsular polysaccharide (CPS) is the most common virulence determinant, which is the basis of the classical Penner serotyping scheme. Notably, particular serotypes may contribute to disease susceptibility (Heikema et al., 2015), and HS19 has been reported to be over-represented in GBS outbreaks (Kuroki et al., 1993; Nachamkin et al., 1998), indicating that the unique feature of the HS19 isolate might play a causative role in GBS induction. Moreover, lipooligosaccharide (LOS) is another important virulence determinant, and molecular mimicry between the structure of *C. jejuni* LOS and human gangliosides is thought to be related to the development of GBS in patients previously infected with this pathogen (Perera et al., 2007). Isolates belonging to the LOS classes A, B, or C harbor genes (such as *cst-II* and *wlaN*) that enable the incorporation of sialic acid into LOS (Nachamkin, 1997; Parker et al., 2005; Müller et al., 2007). In addition to their association with GBS, strains harboring sialylated LOS are also thought to be related to an increased severity of gastroenteritis (Poly et al., 2011).

In addition to virulence determinants, understanding the status of *C. jejuni* drug resistance is also essential for isolate hazard characterization, which could be critical in the instruction of antibiotics clinically, as well as to implement efficient control measures to reduce human exposure to the pathogen. Antibiotic treatment is indispensable if severe or immunocompromised cases occur, with macrolides and fluoroquinolones (FQs) being the first choice of drugs (Mourkas et al., 2019). However, as a naturally competent organism, *C. jejuni* is capable of incorporating exogenous DNA to adapt to antibiotic selective pressure and is spread by the food chain and water (Kashoma et al., 2016). Over the years, increasing rates of *Campylobacter* strains that are resistant to these two antibiotics. The World Health Organization listed fluoroquinolone-resistant *Campylobacter spp.* as one of the six high-priority pathogens for research and development of new antibiotics in 2017 (Tacconelli et al., 2018). Gentamicin (GEN) and tetracycline (TET) have been reported as alternative therapies (Koolman et al., 2015). Nevertheless, these resistant strains have also been found in multiple types of food animal facilities. As a result, *C. jejuni* is increasingly viewed as a reservoir of antibiotic resistance genes in both human medicine and the food supply chain (Mourkas et al., 2019; Hsu et al., 2020), making antimicrobial resistance (AMR) a public health concern. It could be especially dangerous for people with compromised immunity, since drug resistance greatly limits the available therapeutic effects.

Tracking high-risk animal strains will lead to a better understanding of their distribution in the food chain and provides critical information for the development of targeted mitigation strategies to reduce human exposure (Baker et al., 2012; Buchanan et al., 2017). Because campylobacteriosis is often associated with contaminated food products and exposure to animals, whole-genome sequencing (WGS) might become the preferred typing method, and analysis of isolates from various sources should be a major component of studying disease ecology and epidemiology (Park et al., 2020). At present, WGS has been considered the most informative and discriminative typing method to examine the genomic characteristics of *Campylobacter* isolates with high resolution (Davies et al., 2020), allowing for comprehensive phylogenetic analyses of numerous traits associated with virulence or antibiotic resistance (Fiedoruk et al., 2019). Our previous epidemiological research revealed that the GBS maker serotype HS19 was significantly more prevalent in isolates from cattle than other animal populations circulating in China. Thus, the goal of the current study is to investigate the pathogenic potential of HS19 isolates from cattle. WGS was used for isolate characterization, and the phylogenetic relatedness between HS19 isolates from cattle in China and GBS patients worldwide was analyzed. Additionally, antimicrobial resistance profiles and virulence genes were identified *in silico*, while antimicrobial susceptibility and *in vivo C. jejuni* infection assays were conducted to determine the pathogenic phenotypes.

## Materials and Methods

### HS19 *C. jejuni* Isolates

A collection of 14 cattle isolates, characterized as serotype HS19, were involved in this study. These isolates were identified from fecal samples of cattle populations circulating in Jiangsu province and Liaoning provinces, in eastern China, between 2005 and 2019. Cattle is a common source of animal protein in this geographical area, and cattle isolates were sampled from three large-scale cattle farms, which were selected as the suppliers for cattle slaughterhouses. The sampling procedure was approved by the Research Ethics Committee of Yangzhou University. Additionally, five control strains with HS19 from other sources (diarrhea patients, *n* = 2; pet, *n* = 2; chick, *n* = 1) but were also isolated from Jiangsu province were chosen for phylogenetic analysis, and the background information of these isolates was shown in Supplementary Table 1.

As previously described (Zang et al., 2016), *C. jejuni* strains were routinely cultured on *Campylobacter* selective agar base plates (modified CCDA, Preston; Oxoid, United Kingdom) under microaerophilic conditions (5% O_2_, 10% CO_2_, and 85% N_2_) at 42°C for 48 h. The isolates were identified at the *C. jejuni* species level by PCR, and then stored at –80°C in 15% glycerol in brain heart infusion broth until use. A capsule genotyping scheme was exploited for isolates serotyping (Poly et al., 2011).

### Whole-Genome Sequencing

Genomic DNA of cattle isolates was prepared using the TIANamp Bacterial DNA Kit (Tiangen Biotech, Beijing, China) according to the manufacturer’s instructions. DNA was then fragmented to prepare the library and was sequenced using Illumina NovaSeq 6,000 (Illumina, United States) in the Novogene Institution (Tianjing, China). Reads were assembled into contigs and scaffolds using SOAPdenovo v2.04.^1^ Genomes were annotated using Prokka (Seemann, 2014). WGS data were submitted to the Sequence Read Archive (SRA) database in NCBI with the accession number PRJNA725618 animal isolates,^2^ and the SRA-BioSample numbers ranged from SAMN18896327-SAMN18896340 (Supplementary Table 1).

### Molecular Typing

WGS data of *C. jejuni* isolates from cattle were genotyped by *in silico* multilocus sequence typing (MLST)^3^ (Jolley et al., 2004). Through searching ‘‘HS19’’ and ‘‘19,’’ a collection of 79 HS19 isolates were accessed from the PubMLST database with the access date 2021-9-1.^4^ The sources of these isolates from PubMLST database included cattle (3.8%, 3/79), chicken (7.6%, 6/79), GBS patients (17.7%, 14/79), gastroenteritis patients (59.55%, 47/79), human systemic disease (1.3%, 1/79), and human unspecified (10.1%, 8/79). These human isolates were sampled from 11 countries, including the United Kingdom, Netherlands, South Africa, Japan, Thailand, United States, Mexico, Canada, Belgium, Denmark, and China, while the collection year ranged from 1983 to 2018 (Supplementary Table 3).

Housekeeping allelic profiles of cattle isolates were analyzed using the goeBURST algorithm implemented in PHYLOViZ 2.0 (Nascimento et al., 2017), to create a minimum spanning tree (MST), combined with the corresponding data of 79 human isolates. Moreover, two *C. jejuni* HS19 isolates from GBS patients were downloaded from the NCBI database. The population structures of these 14 cattle isolates were compared with 72 isolates from patients with GBS worldwide, which were accessed from NCBI and PubMLST (Supplementary Table 3).

### Homologous Based Phylogenetic Analysis

A total of 62 WGS sequences of *C. jejuni* isolates were selected for homologous analysis (Supplementary Table 1). Phylogenetic relatedness of 14 HS19 cattle isolates was analyzed, combined with two cattle isolates with HS19 in the United States, 43 control isolates with various serotypes from clinical patients worldwide (including HS19 isolates from GBS patients, *n* = 10; HS19 isolates from diarrhea patients, *n* = 9), and three control isolates with HS19 from other animals in China.

To build a phylogenetic tree, homologous genes were screened using OrthoFinder (Levy et al., 2017), 103867 genes (99.1% of total) were assigned to 2306 orthogroups. There were 1390 orthogroups with all species present, and 1132 of these consisted entirely of single-copy genes. Then, a species tree using 1132 orthogroups with a minimum of 100.0% of species having single-copy genes in any orthogroup to construct a phylogenetic tree. The ModelFinder part tested up to 546 protein models, and HIVb+F+R3 was chosen as the best-fit model according to Bayesian statistics criteria (BIC). Finally, a maximum-likelihood-based phylogenetic tree with a bootstrap value of 1,000 iterations was built using the Iq-tree. Table2itol.R was used to generate iTOL annotations from spreadsheet files in R version 3.2.0.

### Lipooligosaccharide Typing and Polymorphisms Analysis of *cst-II*

Cattle isolates of HS19 were characterized using a PCR-based LOS class typing scheme, performed as previously described (Parker et al., 2005). The sequence of gene *cst-II* was extracted from WGS data by get_homologues-3.3.3 (Contreras-Moreira and Vinuesa, 2013), and the 51st amino acid variations of *cst-II* from the clustering orthologous sequences were analyzed using the Clustalw program.

### *In silico* Identification of Anti-bacterial Resistance Genes and Virulence Genes

The genomes of *C. jejuni* isolates (Supplementary Table 1, column 13) were screened for all known resistance and virulence genes using ABRicate v0.8.10 (Park et al., 2020). Anti-bacterial resistance (ABR) genes were identified by a BLASTN comparison against the Resfinder database (Zankari et al., 2012) and Comprehensive Antibiotic Research Database (CARD) database. Point mutations related to antibiotic resistance genes were identified by PointFinder using the pointfinder database (Zankari et al., 2017). Virulence genes were identified by BLASTN comparison against the Virulence Factor Database (VFDB) (Liu et al., 2019). ABRicate classifies the predicted genes based on the proportion of the gene that is covered, and the threshold for identification was taken to be 60% gene identity and 40% sequence coverage.

### Antimicrobial Susceptibility

The antimicrobial susceptibilities of HS19 isolates (cattle isolates, *n* = 14; diarrhea isolates, *n* = 2; GBS isolate, *n* = 1) were measured by the agar dilution method recommended by the Clinical and Laboratory Standards Institute (CLSI) guidelines (CLSI, 2013). *C. jejuni* ATCC 33560 was used as the quality control strain. Briefly, colonies were subcultured on *Campylobacter* selective agar base CCDA agar plates for 24 h and then seeded in Mueller Hinton broth supplemented with 5% sheep blood (Oxoid, Basingstoke, United Kingdom), with known scalar concentrations of the following antibiotics: ciprofloxacin (CIP) (0.03–128 μg/ml), erythromycin (ERY) (0.5–256 μg/ml), gentamicin (GEN) (0.25–256 μg/ml), chloramphenicol (CHL) (0.25–128 μg/ml), florfenicol (FFC) (0.25–128 μg/ml), clindamycin (CLI) (0.06–128 μg/ml), and tetracycline (TET) (0.25–256 μg/ml). Strains were classified as resistant (R), intermediate (I) or susceptible (S) according to MIC breakpoints in CLSI (VET01-A4, 2013).

### IL-10^–/–^ C57BL/6 Murine Infection Model

IL-10^–/–^ C57BL/6 mice (B6.129P2-IL-10tm1Cgn/J) were obtained from Jackson Laboratories (Bar Harbor, ME, United States). A breeding colony was established under specific pathogen-free conditions. Prior to inoculation, fecal samples were collected to confirm the absence of colitogenic bacteria. Mouse genotypes were identified using a PCR assay from Jackson Laboratories with a one-step mouse genotyping kit (Vazyme, Nanjing, China).

In order to assess the enteritis induction ability of cattle isolates, mice of 6–7 weeks old were orally administrated with fresh suspensions of 14 cattle isolates (0.2 ml of 1 × 10^10^ CFU per mouse), 14–15 mice were intragastrically administrated with each HS19 *C. jejuni* isolate. As a positive control, HS19 *C. jejuni* suspension of one GBS strain was also prepared for mouse infection, whereas sterilized phosphate-buffered saline (PBS) was prepared as the negative control. Fecal pellets were collected at 2 WPI (weeks post-inoculation) to confirm *C. jejuni* colonization. Diarrhea-associated clinical symptoms were observed weekly. After 5 weeks of observation, the mice were euthanized, and the intestinal gross pathology of each mouse was scored during necropsy as previously reported: Grade 0 = no gross pathology detected; Grade 1 = thickened wall (TW) or enlarged (ENL) colon or cecum; Grade 2 = TW or ENL colon and cecum; Grade 3 = TW or ENL colon and cecum and bloody feces or luminal contents (Brooks et al., 2017). Moreover, intestinal tissue was harvested to assess pathological lesions by staining with hematoxylin and eosin (HE) (Oh et al., 2017).

## Results

### Multilocus Sequence Typing Profiles of *C. jejuni* HS19 Isolates From Cattle

**FIGURE 1:**
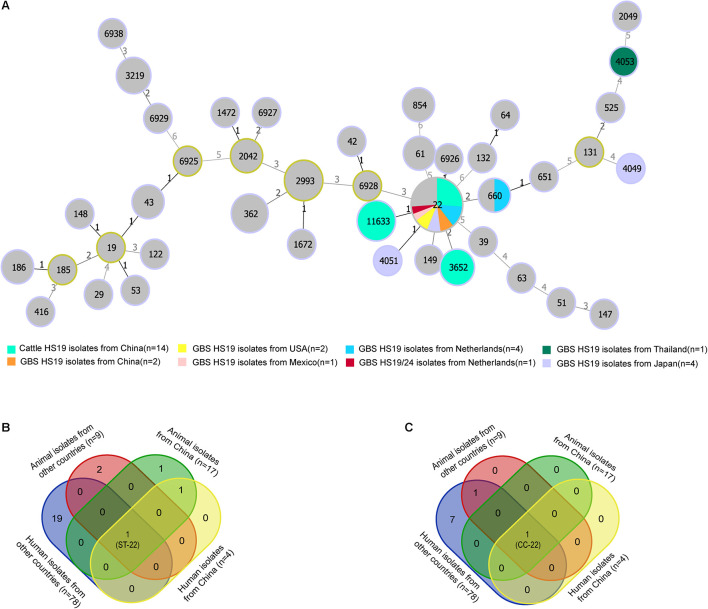
Genotype diversity among *Campylobacter jejuni* HS19 isolates. **(A)** A minimum spanning tree generated for *C. jejuni* HS19 isolates from cattle and Guillain-Barré syndrome (GBS) patients all over the world. The size of the circle proportional to the number of isolates of a particular sequence type. The distance labels correspond to the number of discriminating alleles. GBS isolates with non-HS19 serotypes are represent by gray, whereas HS19 isolates from different geographical locations are represented by other colors. **(B)** Venn diagram shows the sequence type diversities of isolates from human patients and animals. **(C)** Venn diagram shows the clonal complex diversities of isolates from human patients and animals.

MLST analysis revealed restricted genetic diversity for HS19 *C. jejuni* populations, with the ST-22 complex being the most common clonal complex (CC) for both cattle isolates in China and GBS isolates worldwide. A total of 14 cattle isolates of HS19 belonged to three unique known STs (Figure 1A), and all were grouped into the ST-22 complex. ST-22 was represented of 42.86% (*n* = 6) cattle isolates, followed by ST-11633 (35.7%, *n* = 5) and ST-3652 (21.4%, *n* = 3). In contrast, 15 GBS isolates with HS19 belonged to five unique known STs and were grouped into three CCs. Similarly, the most common ST was ST-22 (73.3%, *n* = 11), followed by ST-660 (6.6%, *n* = 1), ST-4051 (6.6%, *n* = 1), ST-4053 (6.6%, *n* = 1), and ST-4049 (6.6%, *n* = 1). CC-22 accounted for 86.6% (*n* = 13) of GBS isolates, which were mainly sampled from the Netherlands (38.4%, *n* = 5), followed by Japan (23.0%, *n* = 3), China (15.3%, *n* = 2), United States (15.3%, *n* = 2), and Mexico (7.6%, *n* = 1). Nevertheless, regarding 71 GBS isolates with various serotypes (Supplementary Table 2), CC-22 was the most common clonal complex (32.3%, *n* = 23). MLST profiles of four collections of *C. jejuni* HS19 isolates from different sources were compared (Supplementary Table 3), including animal isolates from China (*n* = 17), human isolates from China (*n* = 4), human isolates from other countries (*n* = 78), and animal isolates from other countries (*n* = 9). ST-22 (Figure 1B) and ST-22 complex (Figure 1C) were the only sequence type and clonal complex shared by each isolate collection, respectively.

### Phylogenetic Relatedness

**FIGURE 2:**
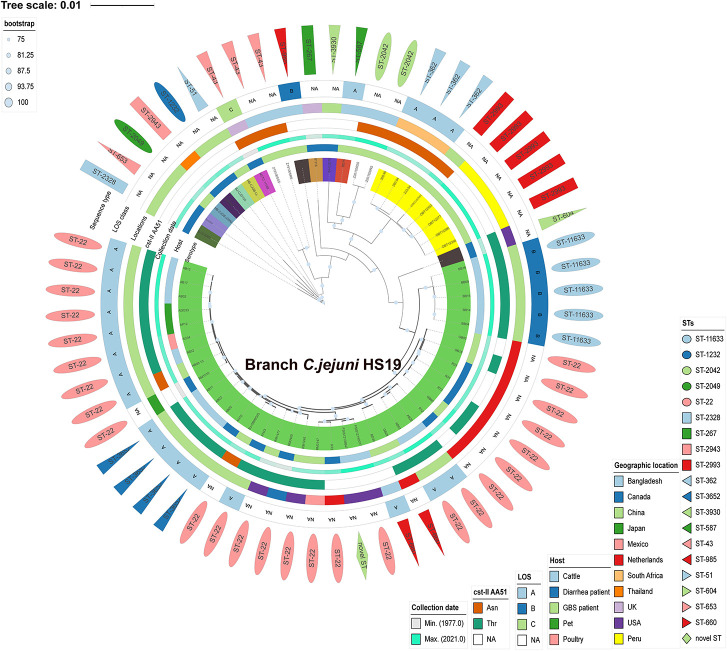
Phylogenetic tree of *Campylobacter jejuni* isolates according to Bayesian statistics. Serotype and genotype are annotated in this tree. The green tree color refers to the HS19 isolates from cattle and human patients. Other tree colors in the innermost circle (covering strain name) refer to the isolates of other serotypes. Next to the tree color, from inside to outside, five different color strips individually reflect the host, collection year, *cst-II* AA51, geographical location, and LOS class. The outermost layer of different shapes refers to various sequence types.

*C. jejuni* HS19 cattle isolates from China showed close phylogenetic relationships with HS19 isolates from GBS patients and diarrhea patients worldwide between 1983 and 2015. Phylogeny was based on orthologous relationships between sixty-two isolates. Overall, the 14 HS19 isolates from cattle in China, together with other 24 HS19 isolates from cattle, poultry, pet, GBS patients, and diarrhea patients worldwide, were grouped into an independent branch marked as “Branch *C. jejuni* HS19” in Figure 2. In contrast, isolates identified of other serotypes were grouped into other branches. Notably, *C. jejuni* HS19 strains from clinical patients included 9 GBS strains and 10 diarrhea strains, and the collection dates ranged from 1980 to 2015. The most dominant geographic location was the Netherlands, followed by China, the United States, Mexico, Canada, Japan, and Bangladesh.

### Analysis of Sialylated Lipooligosaccharide Class and *cst-II* Polymorphisms

Sialylated LOS locus classes were detected in all cattle isolates of HS19 (Supplementary Table 1), including LOS A class (64.2%, 9/14) and LOS B class (35.7%, 5/14). Notably, these cattle isolates shared the 51st amino acid Threonine (Thr) in *cst-II* with six GBS HS19 isolates (the United States, Mexico, and the Netherlands) and four diarrhea isolates (China, the Netherlands, Canada) all over the world, suggesting that these isolates could form a GM1-like ganglioside mimic. Moreover, Asparagine^51^ (Asn) was detected in the *cst-II* gene in two GBS isolates of HS19, which could produce ganglioside mimics residues such as GT1a-like, GD3-like, and GD1c-like LOS.

### Distribution of Anti-bacterial Resistance Genes

**FIGURE 3:**
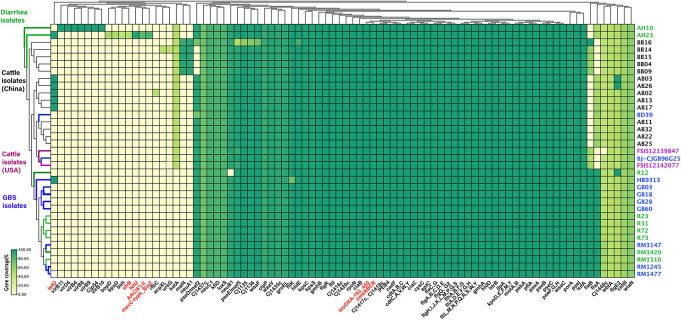
Summary of the antimicrobial resistance genes and virulence profiles of *Campylobacter jejuni* HS19 isolates. The virulence genes and antimicrobial resistance genes are listed at the bottom part of the figure, and colored in black and red, respectively. Names of isolates are listed on the right, in detail, cattle isolates from China are in black, cattle isolates from United States are in purple, diarrhea isolates worldwide are in green, whereas isolates from patients with Guillain-Barré syndrome are in blue. Further, for the small rectangle, green color indicates the presence of a 100% gene sequence coverage, light green indicates the presence of 60% sequence coverage, and yellow indicates the absence of a gene.

The presence of horizontally acquired genes known to encode resistance to a range of different classes of antibiotics among Chinese cattle isolates (HS19, *n* = 14) was determined and compared with the corresponding data of GBS isolates (HS19, *n* = 10), diarrhea isolates (HS19, *n* = 9), and two cattle isolates from the United States (HS19, *n* = 2). A total of four unique ABR genes were identified, representing three different major classes of antibiotics (beta-lactams, tetracycline, *CmeABC* multidrug efflux complex, and *CmeR*) (Figure 3). All of these isolates (100%, 39/39) carried at least seven horizontally acquired resistance genes, including *blaOXA-193*, *blaOXA-450*, *cmeA, cmeB, cmeC*, and *cmeR*, with a gene coverage of 99.99–100% and identity percentage of 95.2–99.87%. *tet(O)* was present in 35.7% (5/14) of cattle isolates and 10.5% of clinical isolates, with a gene coverage of 100% and identity percentage of 99.27–99.74%. The detailed gene identity percentage and sequence coverage of each *C. jejuni* isolate is shown in Supplementary Table 4. Moreover, *gyrA* (T86I) was detected in 62.3% of cattle isolates, and 15.8% of clinical isolates, with a gene coverage of 99.99–100% (Table 1).

**TABLE 1:** Antimicrobial resistance genetic determinants predicted in *Campylobacter jejuni* HS19 isolates.

**Antibiotic class**	**Gene**	**Predicted phenotype**	**Frequency (%)**		
			**Isolate from cattle (*n* = 14)**	**Isolate from GBS patients (*n* = 10)**	**Isolate from diarrhea patients (*n* = 9)**
Tetracyclines	*tet(O)* ^a^	Tetracycline	35.70%	10.00%	11.10%
			(5/14)	(1/10)	(1/9)
	*tet(U)* ^b^	Tetracycline	0.00%	0.00%	11.10%
			(14/14)	(0/10)	(1/9)
CmeABC multidrug efflux complex	*CmeABC* and *CmeR*^a^	Multidrug resistance	100.00%	100.00%	100.00%
			(14/14)	(10/10)	(9/9)
EfrAB efflux pump	*efrB* ^c^	Multidrug resistance	0.00%	0.00%	11.10%
			(0/14)	(0/10)	(1/9)
Aminoglycosides	*aac(6′)-Ii* ^d^	Dibekacin, Gentamicin, netilmicin sisomicin, tobramycin	0.00%	0.00%	11.10%
			(0/14)	(0/10)	(1/9)
β-Lactams	*OXA-193, OXA-450* ^a^	Carbapenem, cephalosporin, penam	100.00%	100.00%	100.00%
			(14/14)	(10/10)	(9/9)
	*mecC-type BlaZ^e^*	Blaz-like beta-lactamase	0.00%	0.00%	11.10%
			(14/14)	(0/10)	(1/9)
Point mutations	*gyrA* (T86I)^f^	Ciprofloxacin I/R, Nalidixic acid	62.30%	10.00%	22.20%
			(9/14)	(1/10)	(2/9)

*^a^tet(O), CmeABC and CmeR, OXA-193,OXA-450, gyrA (T86I): sequence coverage 99.9–00%, gene identity percentage 95.2–99.87%.*

*^*b*^tet(U): sequence coverage 96.9%, identity percentage 77.3%.*

*^*c*^efrB: sequence coverage 47.7%, identity percentage 78.1%.*

*^*d*^aac(6′)-Ii: sequence coverage 78.3%, identity percentage 99.8%.*

*^*e*^mecC-type_BlaZ: sequence coverage 85.0%, identity percentage 67.5%.*

*^*f*^gyrA (T86I): mutation ACA–> ATA (T–> I); coverage 100%, identity percentage 99.9%.*

### Antimicrobial Resistance Phenotypes

**FIGURE 4:**
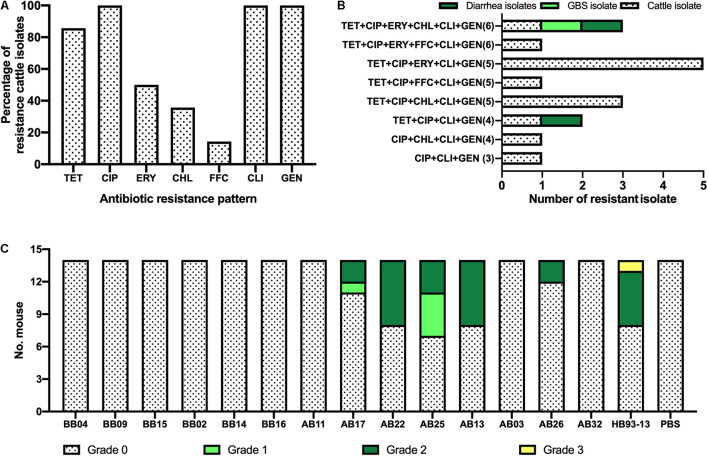
Phenotypic characterization of *Campylobacter jejuni* HS19 isolates from cattle. **(A)** Prevalence of *C. jejuni* antibiotic resistance for seven antibiotics, including ciprofloxacin (CIP), erythromycin (ERY), gentamicin (GEN), chloramphenicol (CHL), florfenicol (FFC), clindamycin (CLI), and tetracycline (TET). **(B)** Number of resistant *C. jejuni* isolates from cattle, Guillain-Barré syndrome (GBS) patients, and diarrhea patients showing multi-drug resistance. **(C)** Gross pathologies of the intestinal tissues induced by the *C. jejuni* isolates from cattle and GBS patients.

Higher levels of AMR in cattle isolates are shown in Figure 4A and Table 2. The most dominant AMR included CIP, CLI, and GEN, all of which were present in all isolates, followed by TET (86%, 12/14), ERY (50%, 7/14), and CHL (36%, 5/14). A lower resistance level was observed for FFC, with a proportional representation of 14% (2/14).

**TABLE 2:** ABR gene and antimicrobial resistance phenotype of *Campylobacter jejuni* HS19 isolates in China.

**Isolate**	**Source**	**Coverage of ABR gene (%)^a^**					**Antimicrobial resistance phenotype^b^**						
		***blaOXA-193***	***blaOXA-450***	***cmeABCR***	***tetO***	***gyrA (T86I)***	**TET**	**CIP**	**ERY**	**CHL**	**FFC**	**CLI**	**GEN**
AB02	Cattle	100	100	100	100	100	R	R	S	R	S	R	R
AB03	Cattle	100	100	100	100	100	R	R	S	R	S	R	R
AB11	Cattle	100	100	100	0	100	S	R	S	R	S	R	R
AB13	Cattle	100	100	100	100	100	R	R	S	R	S	R	R
AB17	Cattle	100	100	100	100	100	R	R	R	S	S	R	R
AB22	Cattle	100	100	100	0	100	R	R	R	S	R	R	R
AB25	Cattle	100	100	100	0	100	S	R	S	S	S	R	R
AB26	Cattle	100	100	100	100	100	R	R	S	S	S	R	R
AB32	Cattle	100	100	100	0	100	R	R	R	R	S	R	R
BB04	Cattle	100	100	100	0	0	R	R	R	S	S	R	R
BB09	Cattle	100	100	100	0	0	R	R	R	S	S	R	R
BB14	Cattle	100	100	100	0	0	R	R	S	S	R	R	R
BB15	Cattle	100	100	100	0	0	R	R	R	S	S	R	R
BB16	Cattle	100	100	100	0	0	R	R	R	S	S	R	R
AH10	Diarrhea patient	100	100	100	0	100	R	R	S	S	S	R	R
AH23^c^	Diarrhea patient	100	100	100	100	100	R	R	R	R	S	R	R
HB93-13	GBS patient	100	100	100	100	0	R	R	R	R	S	R	R

*^*a*^Sequence coverage of the targeted ABR gene in the Card and Resfinder databases. Point mutations related to antibiotic resistance genes were identified using the Pointfinder database.*

*^*b*^TET, Tetracycline; CIP, ciprofloxacin; ERY, erythromycin; CHL, chloramphenicol; FFC, florfenicol; CLI, clindamycin; GEN, gentamicin; R, resistance; S, susceptible.*

*^*c*^The ABR genes only predicted in this diarrhea isolate are not showed in this table.*

Notably, worrying levels of multidrug resistance (MDR) were observed in all cattle isolates. TET+CIP+ERY+CLI+GEN was the most common MDR profile with the highest proportion of MDR in cattle isolates (35.7%, *n* = 5), followed by TET+CIP+CHL+CLI+GEN (21.4%, *n* = 3), CIP+CHL+CLI+GEN (*n* = 1, 7.1%), TET+CIP+ ERY+CHL+CLI+GEN (*n* = 1, 7.1%), TET+CIP+ FFC+CLI+GEN (*n* = 1, 7.1%), TET+CIP+CLI+GEN(*n* = 1, 7.1%), TET+CIP+ERY+FFC+CLI+GEN (*n* = 1, 7.1%), and CIP+CLI+GEN (*n* = 1, 7.1%). In particular, cattle isolates and human clinical isolates shared two kinds of MDR profiles. In detail, TET+CIP+ERY+CHL+CLI+GEN was present in one diarrheal isolate, one GBS isolate, and one cattle isolate, whereas TET+CIP+CLI+GEN was present in another diarrhea isolate and cattle isolate (Figure 4B).

### Distribution of Virulence Determinants

The number of virulence-related genes among the 14 cattle isolates ranged from 106 to 108 (Figure 3 and Supplementary Table 5). Cattle isolates could be divided into two groups based on gene difference; *maf4* and *neuA1* were the two genes shared by five strains, coding motility accessory factor and bifunctional beta-14-N-acetylgalactosaminyltransferase, respectively, which were absent in both the left cattle isolates and disease control isolates. However, another motility accessory factor associated gene, *pseD/maf2*, was absent in these cattle isolates, but was present in the remaining isolates.

The GBS-associated genes *wlaN* and *cst-III* were prevalent among all the cattle isolates, encoding beta-13 galactosyltransferase involved in the biosynthesis of ganglioside-mimicking LOS and lipooligosaccharide sialyltransferase, respectively. Genes *pseA* and *pseI* are required for the biosynthesis and/or transfer of pseudaminic acid to the flagellin, which were also prevalent in all genomes. Other virulence genes shared by each isolate were those encoding traits related to flagella (*flgC et al*), cytolethal distending toxin (*cdtA*, *cdtB*, and *cdtC*), chemotaxis (*cheA*, *cheV*, *cheW*, *cheY*), invasion (*ciaB*, *flaC*), and adhesin (*cadF*, *jlpA*, *porA*, and *pebA*).

Genes unique to a certain strain were observed. *rfbC* was present in a cattle isolate, coding capsule-associated dTDP-4-dehydrorhamnose 35-epimerase, but only blasted with 48.35% coverage. Moreover, *ureG* and *acpXL* were detected in another cattle isolate, coding for the LPS-related acyl carrier protein in *Brucella melitensis bv. 1 str. 16M* and urease accessory protein ureG in *Helicobacter pylori 26695*, individually, but all blasted with 42.17% coverage. Notably, one cattle isolate carried LOS (*Cj1135*, *Cj1136*, *hldE*, *waaF*) and motility accessory factor (*pseD/maf2, pseE/maf5*) associated genes, but showed different gene coverage when compared to other cattle isolates. One cattle isolate from the United States lacked *Cj1440c*. For human GBS strains, the *fliK* gene encoding flagellar hook-length control protein FliK was harbored in one GBS isolate with a gene coverage of 72.18%, while the coverage of other GBS isolates was 99.83%, indicating that *fliK* could not be associated with disease type but could affect disease severity.

### Assessment of Enteritis

Although shared with the same serotype HS19 and sequence type ST-22, *C. jejuni* HB93-13 and 35.7% (5/14) of *C. jejuni* isolates from cattle induced gross pathology (Score > 0), including bloody feces, inflammation of cecum and colon, pathological, whereas PBS was unable to induce severe gastrointestinal pathology (Figure 4C).

**FIGURE 5:**
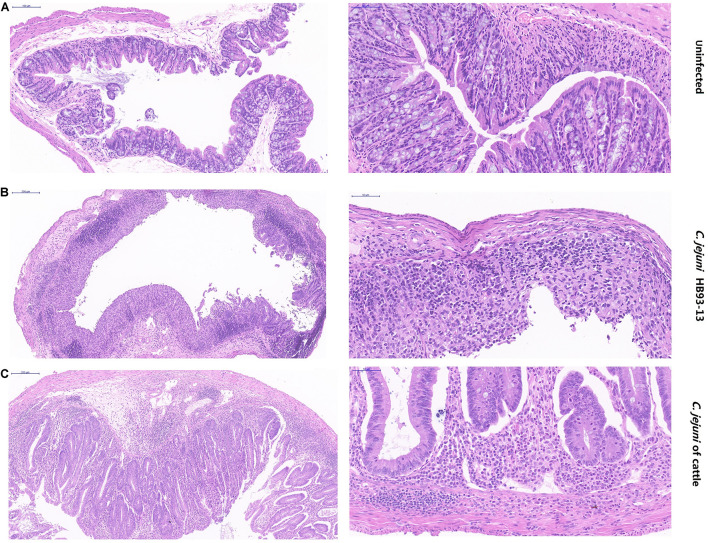
Pathological severity of mouse intestinal tissue. **(A)** Intestinal lesion is not observed in phosphate-buffered saline sham-inoculated mice. **(B)** Human Guillain-Barré syndrome *Campylobacter jejuni* strain HB93-13 inoculated mice showed severe inflammation. **(C)** Cattle isolates induced local inflammatory reaction. Scale bar, 50 and 200 μm.

PBS failed to induce inflammation in the mice (Figure 5A). In stark contrast, serious pathological lesions and inflammation were found in the colon cecum ileum junction (ICCJ), colon, and cecum of mice inoculated with GBS patient isolates and part of cattle isolates. In detail, severe inflammation occurred in mice infected with GBS isolate HB93-13, mucosal epithelial cells were necrotic and exfoliated, and lamina propria contained neutrophils and mononuclear cells (Figure 5B). Mice infected with cattle isolates were also observed with submucosal edema, increased monocytes in the mucous layer, individual intestinal gland necrosis, and local inflammatory reaction spread to the muscular layer (Figure 5C).

## Discussion

Identifying high-risk *C. jejuni* isolates remains an obstacle to effective intervention for campylobacteriosis (Buchanan et al., 2017). Infection of HS19 isolates often increases the risk of developing GBS (Nachamkin et al., 1998), while HS19 isolates are also present in the diarrhea populations circulating in various countries. Since *C. jejuni* colonizes the intestines of various animals, the source of human infection is thought to be a massive reservoir in animal populations (Parker et al., 2005). Based on the idea of “one health” (Wolfe et al., 2007), campylobacteriosis is a zoonotic foodborne disease, and human infection of *C. jejuni* needs to be controlled by animal strains. However, 88.61% of the HS19 isolates in PubMLST database were of human origin. In contrast, zoonotic HS19 isolates have rarely been reported, and the pathogenic potential of *C. jejuni* from animal sources has remained unexplored. In this study, a collection of 14 HS19 cattle isolates from China was systematically characterized both in terms of genotype and phenotype. Remarkably, phylogenetic relatedness and genomic similarity to clinical human isolates were observed among these cattle isolates, as well as a worrying level of multidrug resistance (MDR) and the ability to induce enteritis in a mouse model.

Close phylogenetic relationships between *C. jejuni* isolates from cattle and patients have been frequently reported, although direct epidemiological evidence linking these zoonotic isolates from a specific source as well as a certain disease type was unavailable (Thépault et al., 2017; Hsu et al., 2020). Our results were consistent with those reported ones; cattle isolates of HS19 were observed to have a close genetic relatedness with clinical isolates. Specifically, all of these HS19 isolates obtained from cattle belonged to the ST-22 complex, which has been significantly overrepresented in the isolates among the patients who developed GBS following campylobacteriosis (Nielsen et al., 2010). In addition to GBS, the ST-22 clonal complex has also been reported as a high-risk lineage represented in HS19 isolates, leading to the development of post-infection irritable bowel syndrome (PI-IBS) (Peters et al., 2021). Compared with cattle isolates collected in east China, the majority of human clinical isolates were recovered from developed countries, such as the United States, Netherlands, Japan, and Australia, suggesting the possibility of clonal spread of capsular genotype HS19 isolates across different regions and hosts. Additionally, the predominance of these clonal complexes could be associated with economic conditions, hygiene conditions, wildlife ecology, population movements, and environmental factors (geography and climate) (Oh et al., 2017; Heimesaat et al., 2021). Multiple introductions and widespread dissemination of *C. jejuni* lineages between countries may be facilitated by the constant movement of agricultural products, animals, and people, which could also be associated with the risk of emergence and spread of HS19 isolates, potentially causing pandemics.

Animal microbes and human health are intimately coupled, and animals could be transmitters of high-risk *C. jejuni* isolates to susceptible humans. The sources of HS19 isolates collected in China including cattle, chicken, pets, and human (Supplementary Table 1), while the sources of HS19 isolates from PubMLST database included cattle, chicken, and humans (Supplementary Table 3), indicating HS19 isolates could spread through the food chain “from farm to table.” Thus, in addition to animal types, the prevalence of HS19 isolates could also be influenced by local animal husbandry practices, antibiotic use in farms, transportation of food animals and animal products, and consumption habits. Notably, the collection time also influenced the prevalence of *C. jejuni*. Epidemiological reports from other countries (Habib et al., 2009) suggest that human campylobacteriosis tends to increase between February and September. Therefore, the isolates in this study were sampled during this period.

Identification of virulence factors is crucial to understand the mechanisms of campylobacteriosis infection and to identify if potentially more virulent strains exist (Wysok et al., 2020). Notably, previous studies have identified that genetic determinants are important for *C. jejuni* pathogenicity (Dasti et al., 2010), but they are generally conserved across species. Our results are consistent with the reported ones. Except for the *maf4* coding motility accessory factor and *neuA1* coding bifunctional beta-14-N-acetylgalactosaminyltransferase/CMP-Neu5Ac synthase which were extra represented by five cattle isolates of HS19, other virulence genes harbored in cattle isolates were also represented by human clinical isolates. Notably, even though the cattle isolates from the same collection geography shared the same serotype, ST, AMR genes, virulence genes, as well as similar phylogenetic relatedness with human clinical isolates, only some of the animal isolates could induce the disease phenotype in the mouse model. In fact, although the database of virulence genes is constantly updated, the subject sequence could only be blasted with the reported one, suggesting that unreported pathogenic factors such as accessory genes with a statistically significant difference in carriage rates among animal isolates and human clinical isolates could play a role in *C. jejuni* pathogenicity (Buchanan et al., 2017). Our results also showed that a few genes present both in human isolates and cattle isolates differed in coverage percentage and copy number, which could be a result of the high frequencies of gene transfer and recombination. Besides host susceptibility, gene mutations and polymorphisms could play roles in *C. jejuni* infection, which warrants further investigation.

Increasing rates of *Campylobacter* strains resistant to the drugs of choice and alternative therapies, making AMR *Campylobacter* a public health concern (Mourkas et al., 2019; Gahamanyi et al., 2021), and MDR is still very common in *Campylobacter* strains isolated from farmed animals in many European countries (Pascoe et al., 2017). In this study, MDR was common in *C. jejuni* HS19 isolates from cattle, indicating a potential hazard. TET is often used in the food animal industry because of its low cost and easy administration to animals through drinking water (Jonker and Picard, 2010). Consistent with previous research on a high resistance to TET in China (Zhang et al., 2020), our results showed that *tet(O)* was predicted in 35.7% of cattle isolates, whereas a high resistance level to TET was observed in 86% of cattle isolates. Except for *tet(O)*, only *blaOXA-193*, *blaOXA-450*, *cmeABC*, and *cmeR* were predicted in cattle isolates, although an alarming trend toward MDR was also detected among all cattle isolates, while some of these cattle isolates shared the same MDR profiles with clinical isolates. Notably, 62.3% of cattle isolates were predicted to have point mutations in *gyrA* (T86I), but all of the cattle isolates showed high levels of resistance to CIP. The discrepancies found between the predicted AMR genes and the observed phenotype could be explained by the existence of the efflux pump mechanisms or other unknown resistance mechanisms (Marotta et al., 2020). Moreover, half of the cattle isolates showed resistance to ERY, whereas two clinical isolates showed resistance to ERY. To our knowledge, the use of fluoroquinolones, known to be the first-choice treatment for campylobacteriosis, has recently shifted to erythromycin, against which *Campylobacter* resistance seemed to develop more slowly with respect to fluoroquinolone resistance (Lapierre et al., 2016).

In particular, this study first predicted the *tet(U)* gene in a *C. jejuni* isolate from a patient, with a sequence coverage of 96.86%, and an identity percentage of 77.27%. However, a previous bioinformatic analysis provided compelling evidence that “*tet(U)*” was not a tetracycline resistance determinant, but the misannotated 3′ end of a gene encoding a rolling-circle replication initiator (Rep) protein (Caryl et al., 2012). The potential function of this gene in *C. jejuni* will be investigated in future studies. A few limitations of this study need to be acknowledged. First, the limited sample size of cattle isolates suggested that we have merely touched on the existing genomic diversity of this pathogen. In fact, 14 HS19 cattle isolates and 3 control isolates from other animal sources involved in this study were selected from 1146 animal isolates in a comprehensive genomic epidemiological study, regarding the serotype diversity of *C. jejuni* isolates from various animals in China, within a long sampling time span, since cattle and chicken are the common sources of animal protein, while pets are commonly raised by local citizens. In total, 14 cattle strains of HS19 were identified from 277 cattle isolates. In the future, more cattle isolates will be identified. Another limitation was the underrepresentation of *C. jejuni* isolates from GBS patients and HS19 isolates in the PubMLST database, which needs to be replenished through international cooperation.

Herein, HS19 isolates of animal origin were firstly characterized by a systematic evaluation of bacterial genomics and *in vitro* virulence. Surprisingly, all of the zoonotic isolates belonged to the clinical high-risk lineage with a worrying level of MDR, while the ability to induce enteritis *in vitro* varied among different isolates. Our research is not only a supplement to the HS19 animal isolates in the Public database, but also provides new insight into the pathogenic potential of *C. jejuni* isolates from a putative cattle host. Genetic elements associated with the differences in susceptibility of species as well as the interspecies transmission of these zoonotic isolates to humans should be investigated to advance a better understanding of *C. jejuni*-associated zoonosis.

## Data Availability Statement

The datasets presented in this study can be found in online repositories. The names of the repository/repositories and accession number(s) can be found in the article/Supplementary Material.

## Ethics Statement

Animal experimental procedures were approved by the Laboratory Animal Center of Yangzhou University, according to the experimental animal 3R principle. Animals were housed in the experimental animal center at Yangzhou University, where the environmental conditions meet China’s national standards for environment and facilities for laboratory animals (GB14925-2001). The experimental animal permit license was issued by the Science and Technology Department of Jiangsu Province in China [SYXK (Su) 2017-0044]. Animal experiments were supervised and inspected by the Animal Welfare and Ethics Committee, in accordance with the guidelines of the National Institutes of Health Guide for the Care and Use of Laboratory Animals (NIH publication number 80-23). All mice were euthanized before the autopsy. Animal carcasses, tissues, or body fluids were centralized for pollution-free treatment.

## Author Contributions

XZ: conceptualization, draft preparation, methodology, formal analysis, and visualization. XZ, PH, and JL: investigation. XZ and JH: review. JH and XJ: project administration. JH: funding acquisition. All authors have read and agreed to the published version of the manuscript.

## Conflict of Interest

The authors declare that the research was conducted in the absence of any commercial or financial relationships that could be construed as a potential conflict of interest.

## Publisher’s Note

All claims expressed in this article are solely those of the authors and do not necessarily represent those of their affiliated organizations, or those of the publisher, the editors and the reviewers. Any product that may be evaluated in this article, or claim that may be made by its manufacturer, is not guaranteed or endorsed by the publisher.

## Footnotes

1
https://github.com/aquaskyline/SOAPdenovo2

2
https://www.ncbi.nlm.nih.gov/sra/

3
https://github.com/tseemann/mlst

4
https://pubmlst.org/bigsdb?db=pubmlst_campylobacter_isolates&page=query
